# Mental Health Problems during Pregnancy and the Postpartum Period: A Multicenter Knowledge Assessment Survey among Healthcare Providers

**DOI:** 10.1155/2020/4926702

**Published:** 2020-06-29

**Authors:** M. Patabendige, S. R. Athulathmudali, S. K. Chandrasinghe

**Affiliations:** ^1^Registrar in Obstetrics and Gynaecology, North Colombo Teaching Hospital, Ragama, Sri Lanka; ^2^Registrar in Obstetrics and Gynaecology, Castle Street Hospital for Women, Colombo, Sri Lanka; ^3^Relief House Officer, Castle Street Hospital for Women, Colombo, Sri Lanka

## Abstract

**Background:**

Mental illness related to pregnancy can have long-lasting consequences. Healthcare providers are often the most frequent medical contact with the potential for early detection of these. Objectives were to study the awareness regarding mental health problems during pregnancy and the postpartum period among healthcare providers.

**Methods:**

A cross-sectional study was carried out with healthcare providers including the nursing staff, midwifery staff, and medical officers working at obstetric wards in three tertiary care hospitals in Sri Lanka. A self-administered questionnaire assessed staff experience with mothers having mental problems, knowledge on mental health problems related to pregnancy, and knowledge about risk factors, common symptoms, and possible consequences on a five-point Likert scale from “Strongly Agree” to “Strongly Disagree.”

**Results:**

A total of 300 staff were approached and invited to participate. Only 152 responded to the questionnaire (response rate of 50.1%). Mean (SD) age was 35.8 (9.7) years and mean (SD) years of experience was 10.1 (9.1) years. Age more than 35 years of healthcare providers is associated with statistically significant (*p* = 0.02) average knowledge scores on the consequences of maternal mental health problems. The symptom of “excessively worrying about baby's health” had the lowest score across all three categories with an average of 34.2%. Only 42.8% have ever heard of EPDS. Overall awareness and knowledge about risk factors, symptoms, and consequences regarding pregnancy-related maternal mental health problems are generally good among the healthcare providers studied. However, some of the few aspects are not satisfactory. Health education of pregnant women, promoting regular in-service training sessions, improvement of infrastructure, and involvement of family members from the antenatal period were discussed by the majority.

**Conclusion:**

Despite good overall awareness and knowledge, application into practice with the utilization of validated assessments is poor. This may probably explain why Sri Lanka has a high prevalence of postpartum depression suggesting urgent attention.

## 1. Background

Pregnancy carries a significant risk to the mental health of women. It is vital for all healthcare providers involved in the care of pregnant women including medical practitioners, nurses, and midwives to be well aware of even subtle indicators of maternal mental illnesses and their management options. Postpartum blues are subclinical and transient feelings of fear, anger, and anxiety that affect 50% to 80% of mothers [1]. Although postpartum depression (PPD) is less common, it is by no means a rare occurrence, affecting between 10% and 20% of American women [1]. A study done in 2011 has shown 27.1% of the estimated national prevalence of postpartum depression among Sri Lankan women [2]. It has been reported that about one in three to one in five women in developing countries have a significant mental health problem during pregnancy and after childbirth [3]. Those are mainly depression and anxiety-related problems [3]. There is at least a threefold increase in rates of first onset and severe depression in the postpartum period than in other periods of women's lives [4]. Social factors are important risk factors leading to maternal mental health problems such as poor socioeconomic status, unintended pregnancy, social isolation, and intimate partner violence [5]. So, women in developing countries have a higher propensity for maternal mental health problems in pregnancy and childbirth [5].

Most of these problems are often missed or undertreated, possibly because of its typical features such as fatigue and poor sleep which are also common in motherhood itself [6]. However, it results in many devastating consequences [4, 5]. Those are increased maternal morbidity and mortality with increased risk of maternal suicide and impaired parenting capability which can badly affect the physical, emotional, social, and cognitive development of their children [1, 5]. Also, it can lead to marital problems and future mental health problems. They have an increased risk of obstetric complications and preterm labour [7]. In India and China, suicide is now a leading cause of death in young women in the childbearing age group [5]. One in five pregnant women will experience antenatal depression, and also, these antenatal depressed women have a sixfold increased risk of developing postpartum depression [8].

Early detection and effective interventions where necessary are important to prevent devastating consequences for women themselves, their children, and families. This also reduces the burden of maternal mental health problems for the individual, family, and the entire society. Health professionals working in reproductive health services and caring for pregnant women should be well-trained to recognize symptoms and signs suggestive of a mental health problem and provide effective psychological support and other relevant interventions [5]. A study has shown that the detection of depressive cases was significantly higher with the routine use of this scale (35.4%) by healthcare professionals compared to a control group (6.3%) [9]. Objectives were to study the awareness regarding mental health problems during pregnancy and the postpartum period among healthcare providers working at obstetric wards in three major teaching hospitals in Sri Lanka. The results of this study will help modify maternal health policy interventions with their practical application into clinical practice. These include regular training and awareness programmes, special circulars, and mental health surveillance unit. This may probably help to address 27.1% of the prevalence of postpartum depression in Sri Lanka as aforementioned.

## 2. Methods

### 2.1. Study Design, Setting, and Inclusion Criteria

A hospital-based descriptive cross-sectional study was carried out with healthcare providers including the nursing staff, midwifery staff, and medical officers working at obstetric wards in three tertiary care hospitals in Sri Lanka; Colombo North Teaching Hospital (CNTH), Ragama, Castle Street Hospital for Women (CSHW), Mahamodara (THMG), Galle, Sri Lanka, during 1st of June to 1st of August 2017. In all three hospitals, there were about 300 healthcare providers that are working to provide reproductive healthcare during the study period. All the healthcare providers including all the nursing staff, midwifery staff, and medical officers working at obstetric wards in the above hospitals were invited to participate. Inclusion criteria were the abovementioned categories of healthcare providers working at obstetric wards of CNTH, CSHW, and THMG during the study period.

### 2.2. Procedure

Study instrument was a pretested self-administered anonymous questionnaire consisting of three sections.  Section 1 assessed basic demographic details and experiences with mothers having mental problems.  Section 2 assessed awareness regarding mental health problems related to pregnancy as well as knowledge about risk factors, common symptoms, and possible consequences on a five-point Likert scale from “Strongly Agree” to “Strongly Disagree.” Positively worded and negatively worded items were randomly included in the questionnaire reducing the possible bias.  Section 3 will give space to write down suggestions to improve their knowledge and patient care related to mental illness in pregnancy and the postpartum period. This questionnaire was piloted as a qualitative approach with a group of medical, midwifery, and nursing students who had come for a temporary appointment to an obstetric ward at CNTH. This group was comprised of 15 members, and this group was different from the study population minimizing the bias. Face validity was also carried out for the questionnaire. Investigators carried out the data collection.

### 2.3. Analysis

Data were entered into a data sheet and then analyzed using standard statistical methods. Descriptive statistics were used to analyze nominal data. Chi-squared test was used to see any significant difference between categorical variables. An independent sample *t*-test was applied to see any significant difference between mean knowledge scores between two age categories. A *p* value < 0.05 was considered statistically significant.

Results obtained as answers for section 3 were analyzed qualitatively using thematic analysis. Responses to section three open-ended questions were fully transcribed and analyzed manually. Thematic analysis was done to achieve study objectives. All responses were read several times by the investigators separately to familiarize and to bring out the main ideas and suggestions of participants. Then, discussions among investigators were held to achieve common consensus on the most prevalent ideas expressed in each category. These themes were categorised according to common subtopics. Thematic analysis was performed, and quotations were taken with a common consensus of the group of investigators.

### 2.4. Ethical Considerations

Informed written consent was taken before data collection. Ethical aspects of this study were reviewed, and approval was obtained from the Ethical Review Committee (Reference number: P/77/01/2017), Faculty of Medicine, University of Kelaniya, Ragama, Sri Lanka.

## 3. Results

### 3.1. Quantitative Data

A total of 300 staff members were approached, and all were invited to participate. Only 152 responded to the questionnaire (response rate of 50.1%). Basic demographic and relevant clinical details have been summarized in Table 1. Mean (SD) age was 35.8 (9.7) years, and average (SD) years of experience was 10.1 (9.1). Younger healthcare providers showed a significantly higher (*p* = 0.02) proportion of being heard of EPDS compared to older ones. However, this was not significant (*p* = 0.14) with years of professional experience.

All the “Strongly Agree” and “Agree” answers have been taken as satisfactory answers for the positively worded items and vice versa (“Strongly Disagree” and “Disagree”) for the negatively worded items. Employing that, the number of satisfactory answers for each of the knowledge items under three staff categories was calculated. The average score was calculated as an average (sum divided by the number of knowledge items in each of the four sections) for each of the knowledge item sections, namely, general awareness, risk factors, symptoms, and consequences. These have been summarized in Tables 2 and 3.

Table 2 shows general awareness and knowledge about risk factors for mental health problems during pregnancy and postpartum among healthcare providers. Concerning the general awareness, surprisingly, medical officers showed the lowest awareness compared to the other two categories (*p* = 0.3). Overall, “need for specific medical attention” and “existence of proper medical treatments” for pregnancy-related mental health issues showed a poorer awareness across all three staff categories. Knowledge regarding risk factors was relatively poor for two risk factors across all three categories, namely, “high-risk pregnancies” and “difficult/prolonged labour” with an average score of 64.5% and 66.5%, respectively.

Table 3 shows the knowledge regarding symptoms and consequences of mental health problems during pregnancy and postpartum among healthcare providers. In a nutshell, all three categories of healthcare providers showed a comparable symptom awareness. However, the item on “excessively worrying about baby's health” had the lowest score across all three categories with an average of 34.2%. The item on “tearful and depressed mood” as a symptom was satisfactory only among 54.8% medical officers while it was 85.1% and 82.4% among nurses and midwives, respectively. Overall, knowledge regarding the consequences of maternal mental health problems was satisfactory across all three categories except for the item “adverse effects on the growth of the fetus,” which scored only 55.9% among midwives.

Age more than 35 years of healthcare providers was associated with statistically significant (*p* = 0.02) average knowledge scores on the consequences of maternal mental health problems. Otherwise, there was no significant difference in the remaining three knowledge assessment areas: general awareness (*p* = 0.30), awareness on risk factors (*p* = 0.40), and symptom awareness (*p* = 0.31). There was no significant difference between years of work experience and the average scores of four knowledge assessment areas: general awareness (*p* = −0.34), awareness on risk factors (*p* = 0.66), symptom awareness (*p* = 0.95), and consequences (*p* = 0.19).

For section 3, 69 (45.4%) participants responded and thematic analysis was done for the responses. These can be summarized as follows.

### 3.2. Qualitative Data: Concerns Raised by the Healthcare Workers

#### 3.2.1. Health Education of Women

Health education for pregnant women during the antenatal period regarding mental health problems was expressed by the majority. These included when to seek medical attention and where to get these counseling.

“Give proper advice to pregnant women in general as well as to women at risk of getting mental illnesses especially when to and where to get counseling service and advice. This can lead to early detection of these cases.”

(Medical officer)

#### 3.2.2. Training Programmes and Workshops for the Health Staff for Continuous Professional Development

Regular in-service training sessions and making pregnancy-related mental health problems as one of the core topics to the curriculum of midwifery and nursing programmes were accepted by most of them. Also, improvement of knowledge regarding respectful maternity care, kindness, and maintenance of relevant ethical aspects in good patient care was also described by them as an essential aspect needing attention.

“Updating the knowledge of HCW by in-service training and training of staff to find out socioeconomic background hence to detect liable women. This encourages to advise family members.”

(Medical officer)

“Regular training programmes for the health staff in maternity hospitals can increase the likelihood of detection and timely attention to potential patients.”

(Antenatal ward nurse)

“Training and education on these issues can increase the understanding and sympathy towards mothers who get admitted for the labour and delivery. Moreover, it makes sure kind and optimal care delivery by all levels of HCW.”

(Labour ward midwife)

#### 3.2.3. Involvement of Family Members

Proper education of family members regarding maternal mental health problems, their consequences, and how to detect early can be arranged through field staff and antenatal classes. It was also elaborated that this can yield an idea about women's socioeconomic and family background, helping to detect liable women early.

“Pregnant women need close attention and care from her own family since conception and they need to be kept happy and free from other worries.”

(Antenatal clinic nurse)

#### 3.2.4. Hospital Infrastructure

Setting up of specialist psychiatry units in each maternity hospital was also highlighted. Setting up of psychiatry units in each maternity hospital which makes an easy referral pathway for the potential mothers and arrangement of training sessions for the HCW was indicated by the majority.

“Establishment of psychiatric units with trained staff in every maternity hospital seems to be a timely need and no point of merely researching without implementing these necessities.”

(Labour ward nurse)

## 4. Discussion

This study shows that awareness and knowledge about risk factors, symptoms, and consequences regarding pregnancy-related maternal mental health problems are generally good among the healthcare providers studied. However, three were variations among staff categories. Moreover, the present study showed the health education of pregnant women, promoting regular in-service training sessions, and improvement of infrastructure for better mental health service delivery in maternity hospitals as well as the value of involvement of family members from the antenatal period.

Sri Lanka as a country among other low-middle-income countries is well-known to be a role model in maternal healthcare [10]. However, for the past few years, its rate is hanging around 100 to 120 deaths each year without any significant improvement or worsening [11]. The stagnant maternal mortality ratio over the last decade is a concern in an environment with the almost universal provision of antenatal care and institutional confinements in Sri Lanka. Multiple reasons might have affected this and addressing each factor and area pointed out at the national maternal death surveillance system seems to be a timely intervention. Concerning the common causes of maternal deaths in Sri Lanka, maternal suicides play a common role. An island-wide study found a prevalence of 27.1% of postpartum depression in Sri Lanka [2].

Psychological morbidity among pregnant and postnatal mothers is an important area. The provision of high-quality obstetric care depends on a wide range of structural inputs and effective processes. A recent paper has shown that every year, 25-30 women commit suicide during the pregnancy or within one year after delivery in Sri Lanka [12]. Some of these suicidal deaths are not reflected in the maternal mortality statistics as not all deaths associated with complications of pregnancy, childbirth, and puerperium are classified as maternal deaths if these do not meet the criteria for inclusion [13]. However, despite the criteria, negative impact of maternal death will remain. Researches trying to find out the etiological factors behind the mental health problems in pregnancy and postpartum are a worthwhile effort in this setting.

Recently, the World Health Organisation has announced suicide as a direct cause of maternal death irrespective of the cause [14]. It has been reported that despite frequent contact with healthcare providers during pregnancy and postdelivery, the vast majority of women do not seek help for symptoms of stress, depression, or anxiety or voluntarily disclose their symptoms [15, 16]. This shows the importance of assessing and improving knowledge among healthcare providers. A study has revealed that primary care providers and mental health providers are reluctant to treat pregnant women with depression, may not refer women to alternative sources of care, and may be unaware of risks associated with untreated depression [17].

Another review summarizing obstetrician's views on mental health issues related to pregnancy has identified that they see these issues as important topics [1]. However, it further elaborates that they are not confident in their abilities to diagnose these conditions and are also concerned about the adequacy of their training [1]. Additional training to prepare them to incorporate mental health screening into their practices has been suggested [1]. The present study shows an increased awareness of mental health problems related to pregnancy overall.

In this study, only 42.8% have ever heard of EPDS which is an essential screening tool with regard to the screening of mental health disorders among pregnant and postpartum women [18]. In Sri Lanka, this has been recommended as an essential postnatal screening tool in the maternal care package [19]. This shows that despite overall awareness, application into practice with the utilization of validated assessments is poor. This may probably explain why Sri Lanka has a higher prevalence of postpartum depression suggesting urgent attention [2]. A study has emphasized that time constraints, lack of training, and lack of knowledge of diagnostic criteria are perceived screening barriers for mental illnesses in pregnancy and postpartum [1]. Our study shows that healthcare providers' awareness and knowledge are adequate. But there should be a deficiency in delivering the proper care or identifying the target groups of women in the chain of healthcare reducing suicide-related maternal deaths.

The average score is comparatively lower in some of the items such as the need of specific medical attention (57.9%), the existence of proper treatment (61.2%), higher risk in high-risk pregnancies (64.5%), prolonged labour (66.5%), and excessively worrying about baby's health (34.2%) as shown in Tables 2 and 3. The present study also shows that younger healthcare providers showed a significantly higher proportion of having heard of EPDS compared to older ones. This confers the value of continuous medical education (CME) in this context. The role of improving access and provision of CME programmes has to be thoroughly considered.

In conclusion, this study shows that although awareness and general knowledge seem to be high, several aspects need to be further improved. CME programmes may be helpful. More studies on this topic need to be conducted to address the deficiencies and pitfalls of healthcare providers' service delivery and women's receiving end. This study shows that despite overall awareness, application into practice with the utilization of validated assessments is poor. This may probably explain the higher prevalence of PPD in Sri Lanka. Future research should identify ways to mitigate screening barriers.

### 4.1. Limitations

There are several limitations. The 50.1% response rate is a major limitation due to the self-administered nature of the study. Therefore, it has to be acknowledged that results might have affected by this relatively poor response rate. Since this study is from three teaching hospitals, results may not be generalizable to entire Sri Lanka, although it may give some good insights.

## Figures and Tables

**Table 1:** Basic demographic and relevant clinical details.

Demographic characteristics	Frequency (%), *n* = 152
Center	
NCTH	52 (34.2)
THMG	47 (30.9)
CSHW	53 (34.9)
Designation	
Medical officer	31 (20.4)
Nurse	87 (57.2)
Midwife	34 (22.4)
Age (years)	
≤35	81 (53.3)
36-50	56 (36.8)
51≤	15 (9.9)
Years of experience (years)	
≤5	66 (43.4)
6-10	22 (14.5)
11≤	64 (42.1)
Work setting	
Clinic	10 (6.6)
Antenatal ward	49 (32.2)
Postnatal ward	41 (27.0)
Labour ward	39 (25.7)
Gynaecology ward	12 (7.9)
Ever heard of pregnancy-related mental health problems	152 (100)
Yes	
Dealing with a mother having a pregnancy-related mental health problem during the last 12 months	137 (90.1)
Yes	
Situations in which a mother can get mentally ill	
Antenatal period	3 (2.0)
Postnatal period	8 (5.3)
During labour	0
All the above	135 (88.8)
Ever heard about EPDS	65 (42.8)
Yes	
EPDS can be used in the antenatal period	21 (13.8)
Yes	96 (63.2)
Do not know	
Referring a mother with a pregnancy-related mental health problem for psychiatrist's opinion during the last 12 months	
Yes	84 (55.3)

NCTH: North Colombo Teaching Hospital; THMG: Teaching Hospital, Mahamodara Galle; CSHW: Castle Street Hospital for Women; EPDS: Edinburgh Postnatal Depression Scale.

**Table 2:** General awareness and knowledge about risk factors for mental health problems during pregnancy and postpartum among healthcare providers.

General awareness	Staff category with satisfactory awareness			
	Medical officer, *n* = 31 (%)	Nurse, *n* = 87 (%)	Midwife, *n* = 34 (%)	Overall, *n* = 152 (%)
(1) The way women are getting treated by healthcare providers affects the mother's mental well-being	21 (67.7)	76 (83.4)	29 (85.3)	127 (83.6)
(2) Need of specific medical attention	12 (38.7)	60 (69.0)	16 (47.1)	88 (57.9)
(3) Occurrence of mental illnesses during antenatal period	29 (93.5)	72 (82.8)	30 (88.2)	131 (86.2)
(4) Existence of proper medical treatments	16 (51.6)	56 (64.4)	21 (61.8)	93 (61.2)
(5) Early medical attention is essential	30 (96.8)	85 (97.7)	32 (94.1)	147 (96.7)
Average score (%)	21.6 (69.7)	69.8 (80.2)	25.6 (75.3)	117.2 (77.1)
Risk factor				
(1) High-risk pregnancies	17 (54.8)	60 (69.0)	22 (64.7)	98 (64.5)
(2) History of mental illness in a previous pregnancy	28 (90.3)	81 (93.1)	29 (85.2)	138 (90.8)
(3) Low socioeconomic background	28 (90.3)	76 (83.4)	19 (55.9)	113 (74.3)
(4) Difficult/prolonged labour	19 (61.3)	58 (66.7)	24 (70.6)	101 (66.5)
(5). Bad obstetric history/having a stillbirth/neonatal death in this pregnancy	25 (80.6)	83 (95.4)	30 (88.2)	138 (90.8)
(6) Domestic violence	27 (87.1)	82 (94.3)	33 (97.1)	142 (93.4)
(7) Low self-esteem	27 (87.1)	62 (71.3)	23 (67.6)	112 (73.7)
(8) Unplanned/unnecessary pregnancy	25 (80.6)	76 (83.4)	25 (73.5)	126 (82.9)
Average score (%)	24.5 (79.0)	72.3 (83.0)	25.6 (75.4)	121 (79.6)

**Table 3:** Knowledge regarding symptoms and consequences of mental health problems during pregnancy and postpartum among healthcare providers.

Symptoms	Staff category with satisfactory knowledge on symptoms			
	Medical officer, *n* = 31 (%)	Nurse, *n* = 87 (%)	Midwife, *n* = 34 (%)	Overall, *n* = 152 (%)
(1) Sleep disturbances	28 (90.3)	82 (94.3)	31 (91.2)	141 (92.8)
(2) Tearful and depressed mood	17 (54.8)	74 (85.1)	28 (82.4)	119 (78.3)
(3) Thoughts of self-harm/suicidal ideas	30 (96.8)	84 (96.6)	29 (85.3)	143 (94.1)
(4) Inability to cope with her newborn	28 (90.3)	79 (90.8)	30 (88.2)	137 (90.1)
(5) Excessively worrying about baby's health	17 (54.8)	28 (32.1)	13 (38.2)	52 (34.2)
Average score (%)	24.0 (77.4)	69.4 (79.8)	26.2 (77.1)	118.4 (77.9)
Consequences				
(1) Maternal suicides	30 (96.8)	81 (93.1)	32 (94.1)	143 (94.1)
(2) Adverse effects on growth of the fetus	23 (74.2)	68 (78.2)	19 (55.9)	110 (72.4)
(3) Affects badly on marital life	28 (90.3)	80 (92.0)	32 (94.1)	140 (92.1)
(4) Risk of getting mentally ill in future pregnancies	29 (93.6)	76 (87.4)	27 (79.4)	132 (86.8)
(5) Adverse effects on growth of the newborn	28 (90.3)	73 (83.9)	27 (79.4)	128 (84.1)
Average score (%)	27.6 (89.0)	75.6 (86.9)	27.4 (80.6)	130.6 (85.9)

## Data Availability

Data materials arrived from this study will be available upon a reasonable request from the corresponding author.

## Abbreviations

PPD: Postpartum depression
EPDS: Edinburgh Postnatal Depression Scale
CNTH: Colombo North Teaching Hospital
CSHW: Castle Street Hospital for Women
THMG: Teaching Hospital Mahamodara, Galle
SD: Standard deviation
MMR: Maternal mortality ratio
CME: Continuous medical education.
